# Neuromodulation perception by the general public

**DOI:** 10.1038/s41598-025-89437-8

**Published:** 2025-02-15

**Authors:** Cyril Atkinson-Clement, Andrea Junor, Marcus Kaiser

**Affiliations:** 1 Precision Imaging, School of Medicine, University of Nottingham, Nottingham, UK; 2 NIHR Biomedical Research Centre, University of Nottingham, Nottingham, UK; 3 Department of Neurosurgery, Ruijin Hospital, Shanghai Jiao Tong University School of Medicine, Shanghai, China

**Keywords:** Mental health, Neurology, Treatment adherence, Non-invasive neuromodulation, Surgery, Diseases of the nervous system, Neuroscience, Health care, Medical research

## Abstract

The development of neurotechnologies offers exciting opportunities for novel brain interventions. Public perception plays a crucial role in determining the success and acceptance of these interventions. This study aimed to understand the general non-expert population’s representation of neuromodulation and their preferences for common methods such as pharmaceutical drugs, brain implants, ultrasound, magnetic, and electrical stimulations. We conducted a comprehensive online survey with 784 participants to assess their perception of neuromodulation before and after providing information. We also asked the participants to rank their preferences for different neuromodulation techniques after being provided with information. Statistical analyses included inferential non-linear models and free-text data mining. Our findings revealed that overall, neuromodulation was positively perceived by the participants. Furthermore, providing information resulted in a significant improvement in participants’ perception of neuromodulation. Ultrasound stimulation emerged as the most preferred treatment choice, while pharmaceutical drugs were considered a middle-choice option and brain implants ranked last due to safety concerns. Healthcare providers could benefit from enhancing patient education and awareness to promote informed decision-making and improve treatment adherence. Additionally, stakeholders have to address the existing distrust surrounding pharmaceutical drugs and prioritize the development and promotion of safe, non-invasive neuromodulation treatments.

## Introduction

The ability to manipulate the functioning of the nervous system, known as “neuromodulation,” has inspired both optimistic visions of the future and unsettling tales. While this concept provides entertainment and speculation, it has also spurred the emergence of prominent private companies such as Neuralink© (Fremont, USA) and Synchron© (New York, USA*)* among others. These companies are focused on developing technologies, including brain implants, aimed at enhancing humanity. However, the spotlight on these initiatives has overshadowed other important neuromodulation techniques and objectives, while supplying public imagination. This gap between research realities and public perception is essential for understanding and potentially improving the general population’s acceptance of existing and developing neuromodulation interventions as well as determining which approaches may face rejection.

Not restricted to neuromodulation interventions, therapeutic acceptance leads to improved adherence to treatment, already underscored as a key aspect of clinical efficacy by the World Health Organization^1^. Unfortunately, low treatment adherence is frequent (almost 50% of patients with chronic diseases do not adhere to their treatments^1–3^) and has important consequences (it decreases the clinical benefit of validated treatments^4,5^), leading to many avoidable hospitalizations, disease worsening, and ultimately deaths^6^. Moreover, poor adherence poses challenges from an industrial perspective as it has led to the failure of several promising treatments^7^. The reasons for low adherence are numerous and include, among others, inadequate communication and explanation provided to patients, leading to reduced confidence in treatment efficacy^1,8^ as well as the stigma associated with certain treatments^7^.

Recent advancements in neurotechnology have introduced a new generation of non-invasive neuromodulation techniques that target the brain directly with different energies, including magnetic stimulation (Transcranial Magnetic Stimulation, TMS), electrical stimulation (Transcranial Direct Current Stimulation, TDCS), and ultrasound stimulation (Transcranial Ultrasound Stimulation, TUS). These methods are gaining recognition alongside traditional treatments, such as drugs and implants (mostly used through Deep Brain Stimulation, DBS). However, each approach is perceived differently by the medical community and society. While drugs remain the gold standard treatment for many medical conditions, implants evoke both hope and concern due to their invasive nature and potential risks, even if they often provide better symptom relief than medications. Conversely, non-invasive techniques are less familiar to the public, with TDCS often facing stigma associated with misconceptions regarding electroshock therapy. However, the actual perception of the general non-expert population regarding these approaches remains unknown.

Surveys, particularly online surveys, offer valuable tools for capturing various perspectives related to healthcare interventions. They enable access to diverse populations outside the traditional healthcare system. However, existing surveys often concentrate on assessing treatment efficacy^9^ or soliciting opinions from clinicians^10^. While some surveys address patient perspectives^11^, they generally overlook novel interventions such as non-invasive neuromodulation.

This study aimed to capture the representation of the general non-expert population regarding the general view of neuromodulation, and then compare the five approaches developed above: chemical methods, brain implants, electrical stimulation, magnetic stimulation, ultrasound stimulation. To reduce the complexity and variety of neuromodulation approaches, we only considered methods that target the brain directly (excluding peripherical stimulation such as spinal cord stimulation, vagal nerve stimulation or intrathecal pumps), and we only developed the more common modality of the chosen methods (e.g., magnetic stimulation was presented as a whole category without any distinction between conventional TMS, repetitive TMS, deep TMS, dual coils TMS etc.). We also presented neuromodulation approaches without considering the specific conditions for which they are used, nor any clinical specificity such as their variable effectiveness. This choice was made to achieve two main objectives. First, we aimed to assess how providing information influences people’s general perceptions about what neuromodulation is. This is crucial for understanding the importance of educating patients to demystify brain treatments and enhance their therapeutical adherence. Second, we sought to determine whether certain neuromodulation modalities are preferred or rejected. Although this aspect may not show immediate effects, it could highlight potential directions for future research. For example, recent advancements in temporal interference electrical stimulation demonstrate how research can address early challenges, as this technique can now target deeper areas within the brain.

## Methods

### Participant recruitment

The participants were recruited using a convenience sampling method, which involved reaching out to individuals through various channels, including Twitter/X, UK and international charities focused on brain and mental health conditions, and mailing lists. We did not target a particular population or control for pre-defined criteria. While the majority of participants were from the United Kingdom, we also received responses from individuals around the world, totalling 784 survey completions. Because of the requirement for participants to approve their answers before submission, we were unable to determine the number of people who started but did not finish or submit their answers. Furthermore, since we asked people and institutions to forward our survey to their networks, we could not calculate the total number of individuals who received our invitation or determine the participation ratio.

This online survey was carried out in accordance with relevant guidelines and regulations and was approved by the University of Nottingham Faculty of Medicine and Health Sciences Research Ethics Committee (FMHS 147-1022). Participants confirmed that they were adults (≥ 18 years old) and agreed that their anonymized data would be used for this study in accordance with privacy and data handling standards (GDPR). All participants gave their informed consent to take part in this study.

### Survey questionnaire

The survey underwent several development steps. The first version was developed by researchers’ experts in neuroscience (C.A-C, M.K) in collaboration with neuroscientists and neurologists of the School of Medicine, University of Nottingham. Then, the survey was proofreading by native English speakers and testing on non-expert participants who were unaware of the research objective. The participants took approximately 10 min to complete the survey (median: 10 min and 2 s). The complete questionnaire is included in the supplementary material. The survey consisted of four sections, as outlined below.

The first section provided basic information about neuromodulation (“*Neuromodulation is the alteration of nerve activity through targeted delivery of a stimulus to specific neurological sites in the body. It is carried out to change nervous tissue function*”). Then, participants had to estimate their feelings about this definition by giving a score to 4 positives and 4 negatives adjectives (presented in random order: “*angry*”, “*comfortable*”, “*confused*”, “*excited*”, “*interested*”, “*optimistic*”, “*sad*”, “*worried*”). The scores were obtained on a scale from -5 (“*not [Adjective] at all*”) to +5 (“*very [Adjective]*”). This section aimed to determine how non-expert population perceived the general notion of neuromodulation.

The second section provided more details with the presentation of five different approaches (“*chemical method (drugs)*”, “*brain implants*”, “*electrical stimulation*”, “*magnetic stimulation*”, and “*ultrasound stimulation*”), including their advantages and disadvantages. Then, participants had to rank them based on their preference if, in the future, they had a condition that required them to choose between them. Next, they were asked to provide their degree of perceived effectiveness and safety for all the presented methods. This section aimed to determine how non-expert population perceived each of the general neuromodulation approaches we considered. The same adjectives used during the first step were then presented, and participants had to provide a score on the same scale. Finally, a free-text item was presented for which participants were invited (not mandatory) to declare in a few words what neuromodulation meant to them. This section aimed to determine how the provided information influenced the non-expert population’s perception of the general notion of neuromodulation.

The third section included items to collect information about the participants (sex, age, country and city where they lived, level of qualification, occupation, annual income, regular use of drugs, surgery history, and diagnosis of psychiatric and/or neurological disease). The answer categories were based on the UK Office for National Statistics (ONS) classification.

If the participant reported that they were diagnosed with a psychiatric and/or neurological condition, a fourth section was presented, which included items about the conditions (one or several conditions, classification of the condition [based on the International Classification of Diseases, 11th revision], disease duration, and treatment).

### Statistical analyses

All the statistical analyses were conducted using R. The threshold for significance was set at p ≤ 0.05.

We used χ2 tests to describe our sample. To analyze how neuromodulation was perceived and to rank neuromodulation methods, we used multinomial log-linear tests, which allowed us to preserve the non-linearity of the Likert data distribution. When analyzing the perceived safety and efficacy of all approaches, we utilized linear mixed models. All measured covariables were included in the models to control for them. The reported values were always specific to the covariables and averaged for all the other variables.

To perform word data mining, we collected all words provided by the participants and then applied lemmatization to simplify them. We only selected words that were used by at least two participants and belonged to specific groups, such as adjectives, adpositions, adverbs, interjections, nouns, proper nouns, and verbs. We excluded punctuation, numbers, symbols, and common “*stop words*” (e.g., “*the*”, “*this*”, “*it*”). Subsequently, we conducted multiple binomial regression to determine whether the presence of a specific word was associated with a higher score for a particular adjective. All the adjectives were included in one model to consider any potential collinearity effects.

## Results

### Participant characteristics

In total, 784 individuals participated in the online survey. Table 1 presents the demographic characteristics of the sample along with the observed discrepancies in distribution among the various categories. The analyses revealed an over-representation of females, people living in the UK, people with an educational Bachelor’s level, unemployed or students, and those reporting good health or neurological disorders. Conversely, there is under-representation of individuals aged 75 and older, those from outside the UK, individuals with a Doctoral level of education, workers and office supervisors, and those suffering from psychiatric disorders alone or in combination with neurological disorders. Subsequently, these variables were included in the following analyses to account for these imbalances and mitigate their potential effects.

**Table 1:** Sample characteristics.

Categories	N	%	p-value
**Sex**			< 0.001
Female	544	69.4	< 0.001
Male	223	28.4	0.217
Other/prefer not to respond	17	2.2	< 0.001
**Age**			< 0.001
18–29	162	20.7	1
30–44	199	25.4	0.109
45–59	184	23.5	0.957
60–74	185	23.6	0.844
≥ 75	54	6.9	< 0.001
**Country**			< 0.001
UK	734	93.6	< 0.001
America	18	2.3	< 0.001
Asia	14	1.8%	< 0.001
EU	12	1.5	< 0.001
Oceania	6	< 1	< 0.001
Africa	0	0	< 0.001
**Qualification level**			< 0.001
High school diploma	133	16.9	1
Bachelor’s degree	287	36.6	< 0.001
Master’s degree	164	20.9	1
Doctoral degree	77	9.8	< 0.001
Other	123	15.7	0.257
**Occupation**			< 0.001
Unemployed, students, pensioners, casual workers	311	39.7	< 0.001
Middle management	201	25.6	0.078
Higher management	127	16.2	0.505
Office supervisors	97	12.4	< 0.001
Workers	48	6.1	< 0.001
**Annual income**			< 0.001
Below £13,999	143	18.2	0.474
£14,000–£22,999	127	16.2	1
£23,000–£30,999	90	11.5	1
£31,000–£40,999	110	14	1
£41,000–£62,999	77	9.8	0.093
Above £63,000	77	9.8	0.093
Prefer not to respond	160	20.4%	0.019
**Brain condition**			< 0.001
Healthy	383	48.8	< 0.001
Neurological	279	35.6	< 0.001
Psychiatric	64	8.2	< 0.001
Neurological and psychiatric	58	7.4	< 0.001

p-values were obtained using χ^2^ tests. Red cells correspond to over-representation of the sub-categories while blue cells relate to under-representation.

### Perceptions towards neuromodulation and the effect of providing information

**Fig. 1:**
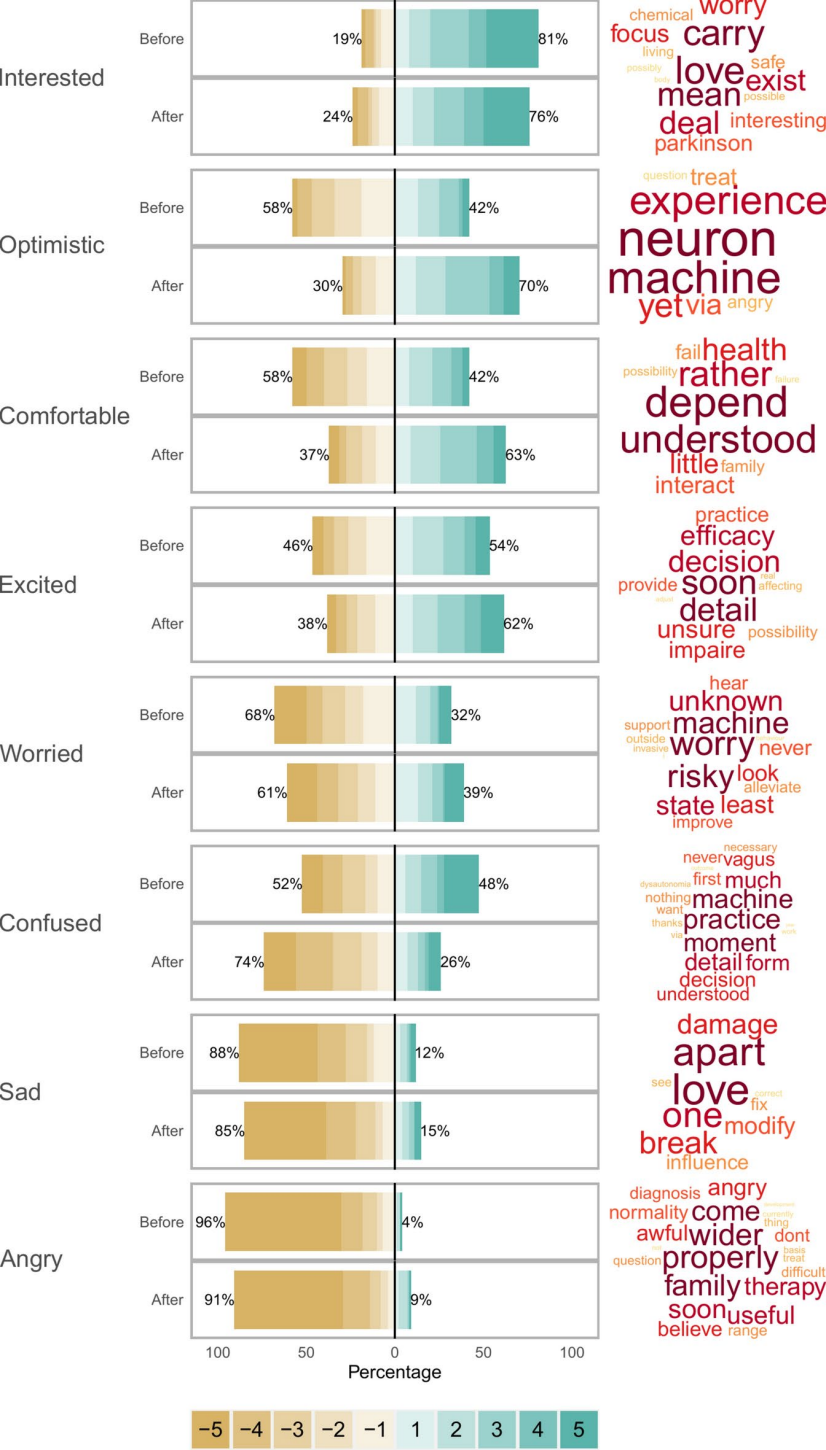
Neuromodulation concept perception. The left panel shows the adjectives we assessed, ranked from the most frequently agree with to the last. The middle panel highlight the degree of agreement for each of the adjectives (after controlling for the effect of all covariables). The scores are provided before and after providing information to the participants. The right panel shows the words significantly and positively related to the adjectives, based on the free-text item. The size of the words is related to the p-value.

We assessed how the general idea of neuromodulation was perceived with only a general definition, and after providing definitions and advantages/disadvantages of the five assessed approaches (Fig. 1 and Table S1). After controlling for other covariables, the concept of neuromodulation was mostly perceived as “*interesting*” (adjusted mean score: +3.01; 81% interested) and did not induce negative feelings except “*confusion*” (adjusted mean score: −0.89; 48% confused). Neuromodulation was not related to any “*optimistic*” feeling (adjusted mean score: −0.09; 58% not optimistic) while the degree of “*worries*” remained high (adjusted mean score: −1.88; 32% worried).

Providing information about the different approaches allowed for a significant increase in the scores of “*optimism*” (adjusted mean difference: +1.64; 70% optimistic), “*comfortable*” (adjusted mean difference: +1.10; 63% comfortable) and “*excitation*” (adjusted mean difference: +0.53; 62% excited). Meanwhile, if the degree of “*confusion*” was improved (adjusted mean difference: -1.21; 74% not confused), the degree of “*worry*” (adjusted mean difference: +0.18; 39% worried) and “*angry*” (adjusted mean difference: +0.05; 9% angry) were slightly worse.

Three group differences were found regarding the difference between healthy participants and patients. Altogether, healthy participants were more negative regarding neuromodulation, with a lower interest (healthy/neurology/psychiatry/both adjusted mean scores: +2.68/ +2.74/ +3.13/ +2.95), and a higher sadness (healthy/neurology/psychiatry/both adjusted mean scores: −3.65/ −3.82/ −3.83/ −3.80) and worry (healthy/neurology/psychiatry/both adjusted mean scores: −1.71/ −1.95/ −1.81/ −1.49). The other effects of the covariables are described in (Table S1).

After giving participants the option to answer questions in their own words and being given information about different techniques, we asked what neuromodulation means for them (“*In just some words, what does neuromodulation mean for you?*”). Using data mining, we extracted a list of 1200-word entities and correlated them (using multiple generalized regression) with the score given to the 8 adjectives after the information was provided. We retained only words for which the correlation was positive (i.e., a higher feeling of [adjective] is related to an increased probability of using this word). Words used by only one participant were discarded.

The “*interested*” adjective was positively related to the use of words such as “*love*” (p < 0.001), “*carry*” (p = 0.005), “*focus*” (p = 0.025), “*exist*” (p = 0.012) and “*deal*” (p = 0.008). Below are two examples from participants with a score of interest =  +5:

*“It could mean the difference between my life going back to normal or existing like this till I die. Nobody wants to be ill and would hopefully jump at the possibility of being “normal” again."* (Male, 44 years old, healthy).

*“A way of treating brain conditions that are currently not well treated. Good news for people struggling with those conditions. Some of the methods sound very risky. Others sound like they are unproven but carry less risk. Some way to go, but interesting.”* (Female, 49 years old, healthy).

“*Optimistic*” was related to words such as “*yet*” (p = 0.007), “*machine*” (p = 0.003), “*neuron*” (p < 0.001), “*angry*” (p = 0.046) and “*experience*” (p = 0.004). Below are two examples from participants with a score of optimistic =  + 5:

*“Interest and intrigue on what’s to come that could help me but also angry that I may not be here to get any benefits from these new neuromodulations. I hope something new that will help will be available soon."* (Male, 75 years old, diagnosed with Parkinson’s disease).

*“To have the possibility to live your life without being conditioned or linked to a pill or a machine.”* (Female, 55 years old, diagnosed with Parkinson’s disease).

The “*comfortable*” adjective was related to the use of words such as “*depend*” (p < 0.001), “*understood*” (p = 0.001), “*family*” (p = 0.034), “*interact*” (p = 0.026) and “*rather*” (p = 0.004). Below are two examples from participants with a score of comfortable =  +5:

*“It sounds like a really intensely useful treatment. I think it would change lives."* (Female, 25 years old, healthy).

*“It’s part of a future where we can use less drugs pumping through our system, causing more side effects and ending up taking more meds to counteract those. We need to get out of this loop.”* (Male, 45 years old, diagnosed with personality disorders and disorders of autonomic nervous system).

“*Excited*” adjective was positively related to the use of words such as “*soon*” (p = 0.001), “*detail*” (p = 0.002), “*practice*” (p = 0.011), “*efficacy*” (p = 0.004) and “*decision*” (p = 0.004). Below are two examples from participants with a score of excited =  + 5:

*“It is future.”* (Male, 34 years old, healthy).

*“The possibility of a treatment for the disease with fewer side effects, greater efficacy and longer lifetime than drugs.”* (Male, 68 years old, diagnosed with Parkinson’s disease).

The “*worried*” adjective was positively related to the use of words such as “*machine*” (p < 0.001), “*risky*” (p < 0.001), “*worry*” (p < 0.001), “*unknown*” (p = 0.009) and “*state*” (p = 0.011). Below are two examples from participants with a score of worried =  +5:

*“Dangerous treating people like guinea pigs.”* (Female, 24 years old, healthy).

*“Too risky, too many dangers and side effects. Too much unknown.”* (Female, 53 years old, healthy).

The “*confused*” adjective was positively related to the use of words such as “*machine*” (p < 0.001), “*practice*” (p < 0.001), “*understood*” (p < 0.001), “*detail*” (p < 0.001) and “*decision*” (p < 0.001). Below are two examples from participants with a score of confused =  + 5:

*“I just hope that I will never be in a position of having to choose for real!"* (Female, 75 years old, healthy).

*“If it could improve daily life and was tested thoroughly, maybe?”* (Female, 58 years old, diagnosed with disorders of autonomic nervous system).

The “*sad*” adjective was positively related to the use of words such as “*apart*” (p = 0.001), “*love*” (p < 0.001), “*one*” (p = 0.005), “*break*” (p = 0.011) and “*influence*” (p = 0.034). Below are two examples from participants with a score of sad =  +5:

*“Apart from the non-invasive treatments, my first reaction is one of disappointment and really horror that as patients, we have so little choice of treatments on the menu. I have met only one Parkinson’s sufferer who has had deal brain stimulation treatment. She has to repeatedly attend hospital to ‘tweak’ electrodes as she continues to suffer profound Parkinson’s symptoms.”* (Female, 70 years old, diagnosed with Parkinson’s disease).

*“The horrible side effects of antipsychotic medication and the uncaring way they are prescribed I’d rather check my lock 3 times a day to make sure goblins aren’t picking the lock than take them.”* (Female, 20 years old, diagnosed with primary psychotic disorders and disorders of autonomic nervous system).

The “*angry*” adjective was positively related to the use of words such as “*soon*” (p < 0.001), “*properly*” (p < 0.001), “*awful*” (p < 0.001), “*diagnosis*” (p < 0.001) and “*angry*” (p < 0.001). Below are two examples from participants with a score of angry =  + 5:

*“To me it is using some form of device or medication to interfere with the nervous system linked normally to some form of brain disease."* (Female, 56 years old, diagnosed with postural orthostatic tachycardia syndrome).

*“Debilitating life. Chronically sick. Unable to live normally.”* (Female, 28 years old, healthy).

### Perception of different neuromodulation techniques

**Fig. 2:**
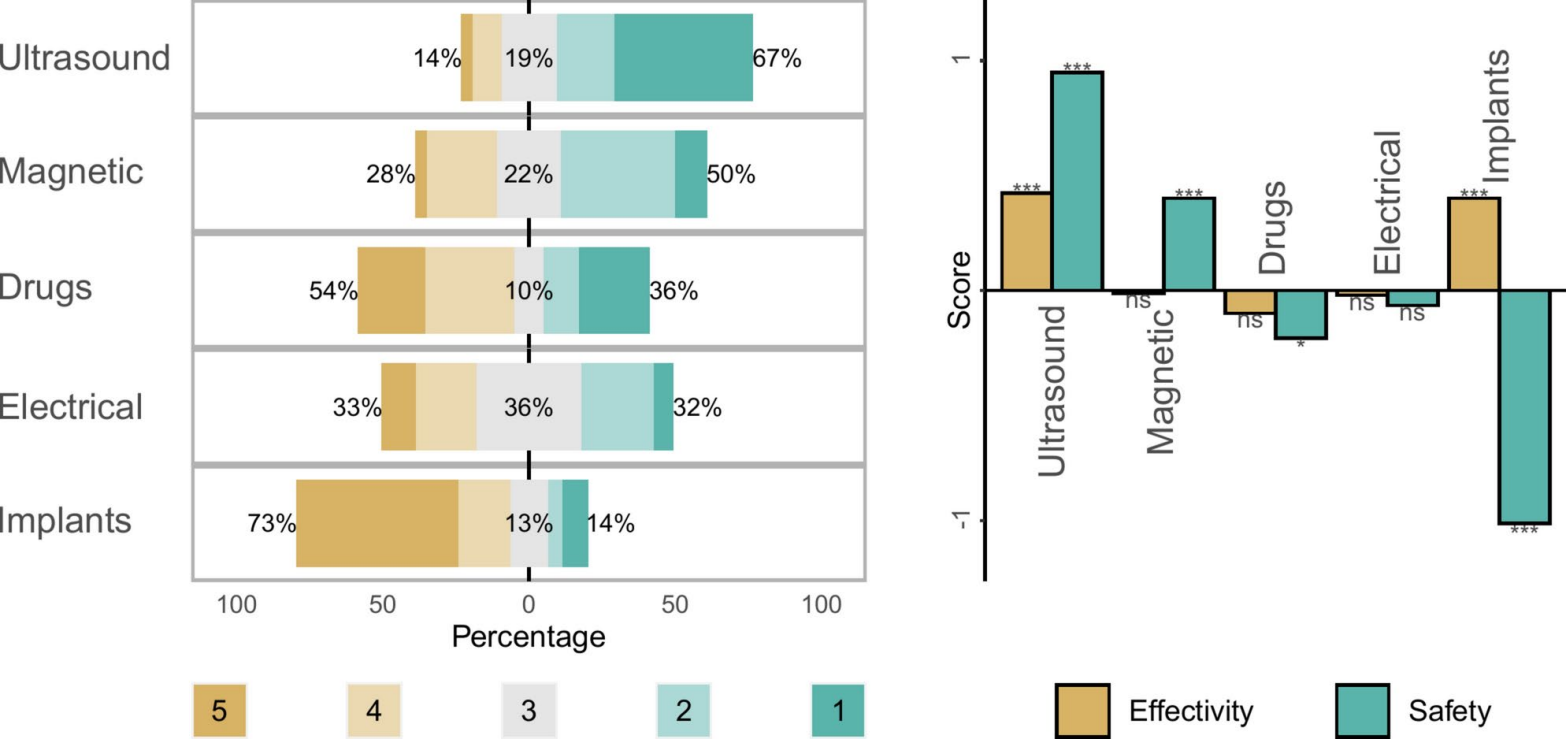
Neuromodulation techniques perception. The left panel shows the ranking for the five assessed neuromodulation techniques, from 1 (first choice) to 5 (5th choice). The scores are averaged for all other covariables. The percentages correspond to the low-ranking (5 an 4th positions), middle-ranking (3rd position) and high-ranking (2nd and 1st positions). The right panel shows the mean scores of perceived safety and efficacy for the five techniques. *ns* not significant; *p-value < 0.05; **p-value < 0.01; ***p-value < 0.001.

In the second step of analyses, we focused on how the five assessed neuromodulation techniques were perceived. We first asked the participants to rank approaches from first to last position (“*In the future, if you have a condition which requires you to choose between these approaches, what would be your preferences? (can you rank them from your first to your last choice?)”*), and we then asked them to give a score of perceived safety and efficacy (from -3 [“*Not safe/effective at all*”] to +3 [“*Totally safe/effective*”]). The analyses were performed using multinomial log-linear tests for the methods ranking and linear mixed models for the scores of safety and effectivity (see Fig. 2, Tables S2–S4).

After controlling for other covariables, we found that ultrasound stimulation was the most frequently ranked at first or second position (67%), followed by magnetic stimulation (50%), drugs (36%), electrical stimulation (32%) and brain implants (14%). No influence of clinical subcategories was observed in this ranking. Conversely, we found that drugs were preferred by young people (18–29 years old), brain implants by people with a high school educational level, magnetic stimulation by males and ultrasound stimulation by females.

With regard to perceived safety (controlled for other covariables), ultrasound (adjusted mean score: +0.947, p < 0.001) and magnetic stimulations (adjusted mean score: +0.401, p = 0.001) were perceived as safe, whereas no clear opinion was found for electrical stimulation (adjusted mean score: −0.065, p = 0.523). Finally, drugs (adjusted mean score: −0.207, p = 0.044) and brain implants (adjusted mean score: −1.013, p < 0.001) were mostly perceived as unsafe.

For perceived effectivity (controlled for other covariables), only ultrasound (adjusted mean score: +0.423, p < 0.001) and brain implants (adjusted mean score: +0.400, p < 0.001) were considered effective, with no clear opinions for magnetic (adjusted mean score: −0.011, p = 0.906) and electrical (adjusted mean score: −0.020, p = 0.834) stimulations, as well as for drugs (adjusted mean score: −0.098, p = 0.315).

## Discussion

We conducted a comprehensive and extensive study involving 784 participants to examine their perceptions of various neuromodulation techniques, including pharmaceutical drugs, brain implants, magnetic stimulation, electrical stimulation, and ultrasound stimulation. Our thorough analysis, accounting for multiple covariables, uncovered several remarkable findings: (i) Overall, participants demonstrated a positive perception of neuromodulation. (ii) Interestingly, participants exhibited significantly increased optimism and decreased negative perceptions when provided with information, emphasizing the pivotal role of education and awareness in understanding emerging medical interventions. (iii) Among the various techniques studied, ultrasound stimulation has emerged as the most favored treatment option, primarily due to its perceived safety and effectiveness, thus underscoring its potential as a promising avenue for further research and clinical application. (iv) Pharmaceutical drugs were perceived as a middle-choice option, with participants expressing doubts regarding their safety and effectiveness. (v) While perceived as highly effective, brain implants ranked lowest in terms of safety. (vi) Our analysis indicated minimal significance regarding the covariables, suggesting that perceptions of neuromodulation techniques are largely consistent across the general non-expert population.

This study represents a significant advancement in the field due to its inclusion of a large and diverse participant sample. By encompassing a comparatively high number of participants combined with multifactorial data analyses, our study provides robust and generalizable insights into the perceptions of neuromodulation techniques in the general population. Another strength of our research is the comparison between different techniques, while most of the research is focused on only one.

Despite these strengths, some limitations warrant acknowledgement. First, we used a convenience sampling method, which may have introduced bias and resulted in an unbalanced participant sample. Typically, surveys use quota sampling, allowing direct extrapolation of the results observed in a sample. The method we used required statistical analysis to be meaningful, and this is what we did to mitigate this concern through rigorous statistical analyses, wherein all recorded covariables were integrated into the models. Consequently, the effects reported in our study were averaged across these covariables, minimizing the impact of participant characteristics on our findings. Second, the manner in which we provided information to participants regarding each neuromodulation approach may have influenced our results. While this aspect introduces a potential source of bias, it also underscores the significance of communication in shaping the perceptions of neuromodulation.

Our study uncovered a significant finding regarding how providing information can impact people’s perception of neuromodulation. The data showed that, after receiving information, participants displayed a notable increase in optimism (+1.64), comfort (+1.1), and excitement (+0.53). At the same time, there was a noteworthy decrease in confusion (−1.21), indicating a clearer understanding of neuromodulation. Although there was a slight increase in worry (+0.18) and anger (+0.05) after receiving the information, the change was minimal. Overall, this suggests that the informational intervention had a predominantly positive effect on participants’perceptions.

These findings have significant implications for treatment adherence, which is crucial for public health, as highlighted by the World Health Organization^1^. Poor treatment adherence has been linked to decreased treatment success^12^, emphasizing the importance of addressing barriers to adherence, such as inadequate knowledge about treatments^13^. This is especially relevant since the Internet makes it easy to access a high range of content regarding treatments that are usually not verified for correctness^14^ and could be misleading^15^. Consistent with the existing literature, our results indicate that providing patients with adequate knowledge of treatments plays a critical role in treatment adherence^16^. Informational interventions have the potential to bolster treatment adherence rates by enhancing understanding and fostering positive perceptions of neuromodulation. Furthermore, other studies have reported interesting findings regarding therapeutic goals from the patient’s perspective^17^ and their post-treatment experiences^18^.

Our analyses uncovered a notable preference for non-invasive neuromodulation approaches, particularly ultrasound and magnetic stimulation, over pharmaceutical drugs and brain implants. This preference aligns with the perceived safety associated with non-invasive techniques, despite lower perceived efficacy, especially in the case of magnetic stimulation. The preference for non-invasive methods over more invasive alternatives echoes the findings from previous research, where methods expected to carry a lower probability of side effects tend to be favored. This phenomenon is consistent with broader trends in healthcare decision-making, where psychological treatments are often preferred to pharmacological interventions^19^, and pharmacological interventions are preferred to surgical procedures, even when the perceived effectiveness varies^20^. It is also crucial to contextualize treatment efficacy within the framework of treatment preferences. Recent studies have highlighted the fundamental role of treatment preference in adherence, and by extension, treatment efficacy. Indeed, a meta-analysis revealed that treatment preference forms the foundation of treatment adherence^21^, directly influencing the treatment outcomes.

In light of these findings, providing patients with information about various treatment options and allowing them to choose could serve as a promising strategy for improving treatment outcomes. By empowering patients to make informed decisions aligned with their preferences, healthcare providers can enhance treatment adherence, and consequently, treatment efficacy.

In conclusion, our study provides valuable insights into the perception of neuromodulation techniques for clinical use. This highlights several key findings: neuromodulation is generally positively perceived, particularly when applied in clinical contexts. Non-invasive approaches are preferred over invasive alternatives, except for electrical stimulation, which has faced significant stigma since the One Flew Over the Cuckoo’s Nest movie (1975^22^). Pharmaceutical drugs are generally viewed negatively because of concerns regarding their side effects.

These results could have major implications at different levels. The study underscores the importance of providing information to patients as a prerequisite for achieving high treatment adherence and desired outcomes. This should be considered, particularly when the patient’s preferences do not align with clinically relevant options. For example, DBS is more appropriate for patients suffering from Parkinson’s disease, while TMS is more relevant for treating depression. By enhancing patient education and awareness, healthcare providers can promote informed decision-making and improve treatment adherence. There is a clear opportunity for government and industry stakeholders to prioritize the development and promotion of safe and non-invasive neuromodulation treatments. As an example, the recent developments in temporal interference electrical stimulation demonstrate how research into specific neuromodulation modalities can help overcome initial limitations, with electrical stimulation now able to reach deep targets. Addressing the existing distrust surrounding pharmaceutical drugs may further enhance treatment adherence and overall healthcare outcomes. Our insights into the priorities and preferences of current and potential future treatments can inform decisions regarding future research directions for novel interventions. By aligning research efforts with patient preferences and clinical needs, researchers can optimize the development and implementation of innovative neuromodulation techniques.

## Supplementary Information

Supplementary Information.

## Data Availability

The dataset and codes used in the current study are available from the corresponding author upon reasonable request.
